# Understanding the nebulisation of antibiotics: the key role of lung microdialysis studies

**DOI:** 10.1186/s13054-024-04828-z

**Published:** 2024-02-19

**Authors:** Jayesh Dhanani, Jason A. Roberts, Antoine Monsel, Antoni Torres, Marin Kollef, Jean-Jacques Rouby, the European Investigators Network for Nebulized Antibiotics in Ventilator-associated Pneumonia, Kostoula Arvaniti, Mona Assefi, Matteo Bassetti, Stijn Blot, Matthieu Boisson, Adrien Bouglé, Jean-Michel Constantin, Jayesh Dhanani, George Dimopoulos, Jonathan Dugernier, Pauline Dureau, Timothy Felton, Marin Kollef, Antonia Koutsoukou, Anna Kyriakoudi, Pierre-François Laterre, Marc Leone, Victoria Lepère, Gianluigi Li Bassi, Xuelian Liao, Olivier Mimoz, Antoine Monsel, Girish B. Nair, Michael Niederman, Lucy B. Palmer, Paolo Pelosi, Jose Manuel Pereira, Konstantinos Pontikis, Garyphalia Poulakou, Jérôme Pugin, Chuanyun Qian, Jie-ming Qu, Jordi Rello, Jason Roberts, Jean-Jacques Rouby, Christina Routsi, Gerald C. Smaldone, Antoni Torres, Melda Türkoğlu, Tobias Welte, Michel Wolff, Xia Jing, Li Yang, Ting Yang, Ying-gang Zhu

**Affiliations:** 1 Faculty of Medicine, University of Queensland Centre for Clinical Research, Brisbane, Australia; 2 Department of Intensive Care Medicine, Royal Brisbane and Women’s Hospital, Brisbane, Australia; 3 Herston Infectious Diseases Institute (HeIDI), Metro North Health, Brisbane, Australia; 4 Division of Anaesthesiology Critical Care Emergency and Pain Medicine, Nîmes University Hospital, University of Montpellier, Nîmes, France; 5 Unité Mixte de Recherche (UMR)-S 959, Immunology-Immunopathology-Immunotherapy, Paris, Institut National de la Santé et de la Recherche Médicale (INSERM), Hôpital Pitié-Salpêtrière, Assistance Publique-Hôpitaux de Paris, Paris, France; 6 Sorbonne University, GRC 29, Assistance Publique Hôpitaux de Paris (AP-HP), DMU DREAM, Multidisciplinary Intensive Care Unit, Department of Anaesthesiology and Critical Care, Pitié-Salpêtrière Hospital, Paris, France; 7 Department of Pneumology, Institut Clinic del Tórax, Hospital Clinic of Barcelona – Institut d’Investigacions Biomèdiques August Pi I Sunyer (IDIBAPS), SGR 911- Ciber de Enfermedades Respiratorias (Ciberes), University of Barcelona, Barcelona, Spain; 8 Division of Pulmonary and Critical Care Medicine, Washington University School of Medicine, St. Louis, MO USA

**Keywords:** Antibiotic nebulisation, Lung microdialysis, Epithelial lining fluid, Nebulised aminoglycosides, Nebulised polymyxins, Ventilator-associated pneumonia

## Abstract

**Background:**

Nebulisation of antibiotics is a promising treatment for ventilator-associated pneumonia (VAP) caused by multidrug-resistant organisms. Ensuring effective antibiotic concentrations at the site of infection in the interstitial space fluid is crucial for clinical outcomes. Current assessment methods, such as epithelial lining fluid and tissue homogenates, have limitations in providing longitudinal pharmacokinetic data.

**Main body:**

Lung microdialysis, an invasive research technique predominantly used in animals, involves inserting probes into lung parenchyma to measure antibiotic concentrations in interstitial space fluid. Lung microdialysis offers unique advantages, such as continuous sampling, regional assessment of antibiotic lung concentrations and avoidance of bronchial contamination. However, it also has inherent limitations including the cost of probes and assay development, the need for probe calibration and limited applicability to certain antibiotics. As a research tool in VAP, lung microdialysis necessitates specialist techniques and resource-intensive experimental designs involving large animals undergoing prolonged mechanical ventilation. However, its potential impact on advancing our understanding of nebulised antibiotics for VAP is substantial. The technique may enable the investigation of various factors influencing antibiotic lung pharmacokinetics, including drug types, delivery devices, ventilator settings, interfaces and disease conditions. Combining in vivo pharmacokinetics with in vitro pharmacodynamic simulations can become feasible, providing insights to inform nebulised antibiotic dose optimisation regimens. Specifically, it may aid in understanding and optimising the nebulisation of polymyxins, effective against multidrug-resistant Gram-negative bacteria. Furthermore, lung microdialysis holds promise in exploring novel nebulisation therapies, including repurposed antibiotic formulations, bacteriophages and immunomodulators. The technique's potential to monitor dynamic biochemical changes in pneumonia, such as cytokines, metabolites and inflammation/infection markers, opens avenues for developing theranostic tools tailored to critically ill patients with VAP.

**Conclusion:**

In summary, lung microdialysis can be a potential transformative tool, offering real-time insights into nebulised antibiotic pharmacokinetics. Its potential to inform optimal dosing regimen development based on precise target site concentrations and contribute to development of theranostic tools positions it as key player in advancing treatment strategies for VAP caused by multidrug-resistant organisms. The establishment of international research networks, exemplified by LUMINA (lung microdialysis applied to nebulised antibiotics), signifies a proactive step towards addressing complexities and promoting multicentre experimental studies in the future.

## Introduction

Nebulisation of antibiotics has emerged as a promising treatment for hospital-acquired pneumonia and is recommended for ventilator-associated pneumonia (VAP), caused by multidrug-resistant organisms [1, 2]. Effective antibiotic concentrations at the site of infection, the interstitial space fluid (ISF), are crucial for improving clinical outcomes. Nebulised antibiotic delivery can be measured in vitro*,* at the tip of the endotracheal tube. In vivo, nebulised antibiotic delivery can be assessed in epithelial lining fluid, and whole lung tissue homogenate obtained from post-mortem lung biopsies. Epithelial lining fluid techniques risk proximal airway contamination [2]. Tissue homogenates provide antibiotic concentrations reflecting a mixture of distal bronchiole and lung parenchyma concentrations [3, 4]. Neither epithelial lining fluid nor tissue sampling allows for longitudinal pharmacokinetic analyses. This article explores the potential of lung microdialysis to fulfil the unmet need for optimising the nebulised antibiotics delivery.

## Background

Class of antibiotics, nebuliser types, interface in spontaneously breathing patients, type of pneumonia, regional lung aeration, severity of lung infection, modes of ventilation (spontaneous or mechanical) and ventilator settings in ventilated patients affect aerosol delivery [2, 5–7]. Using simulated adult/paediatric mechanical ventilation model, nebulised antibiotic delivery is measured at the tip of the endotracheal tube. In vivo, antibiotic delivery can be assessed in epithelial lining fluid, and whole lung tissue homogenate, or indirectly assessed, using imaging techniques [8, 9]. As the bronchoscope is contaminated in proximal airways during the bronchoalveolar lavage, the resulting epithelial lining fluid concentrations of antibiotics likely represent bronchial concentrations than that of ISF [10] and do not provide longitudinal pharmacokinetic data. Tissue homogenates obtained from lung biopsies provide the average concentration of distal bronchiole, intracellular and ISF compartments. Imaging techniques allow the assessment of aerosolised particles lung distribution but do not provide ISF concentrations [11]. Lung microdialysis is the only technique allowing real-time lung ISF antibiotic concentrations measurements.

### Lung microdialysis

**Fig. 1:**
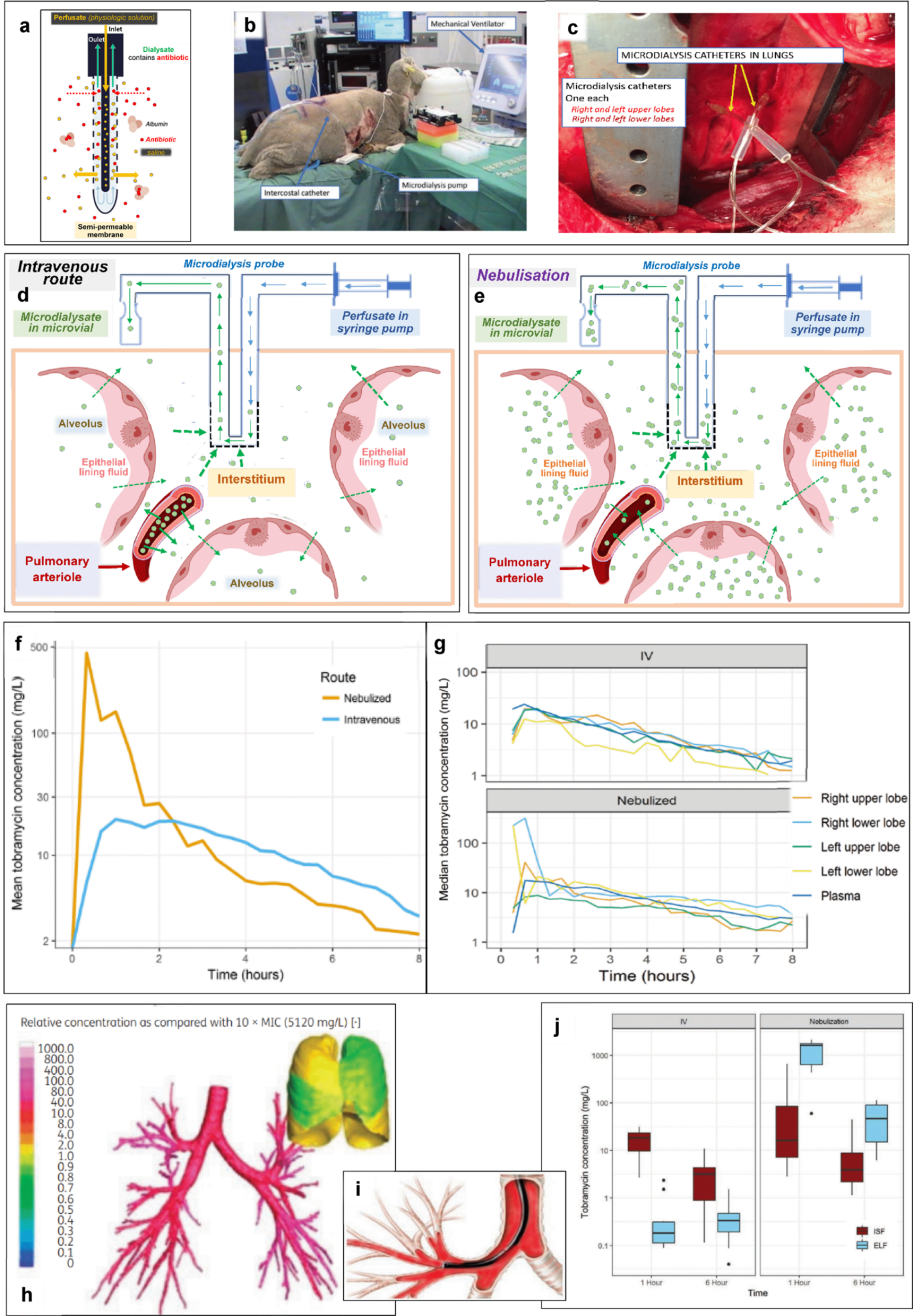
Lung microdialysis for nebulised antibiotics: principles, technique of implementation, assessment of interstitial antibiotic concentrations, advantages over epithelial lining fluid concentrations. **a** A microdialysis probe (0.6 × 50 mm) with a semi-permeable membrane is positioned into the lung parenchyma. A physiologic solution is flushed through the probe using a microdialysis pump (saline yellow filled circle at a flow rate of 0.1–10 µL/min) and the unbound fraction of the antibiotic (red filled circle) present in the interstitium diffuses through the semi-permeable membrane (proteins cannot pass through the membrane). The collected microdialysate containing the antibiotic is analysed by liquid chromatography tandem mass spectrometry; **b** and** c** after thoracotomy, microdialysis probes are inserted under direct vision in the upper and lower lobes of anaesthetised ewes. An intercostal catheter is placed on each side, after incision closure;** d** and **e** combined lung and intravascular microdialysis allows estimation of intravenous and nebulised unbound antibiotics concentrations in the lung and intravascular compartments. As colistimethate sodium (polymyxin E) (green filled circle) has a limited endothelial diffusion, its interstitial and alveolar antibiotic concentrations are low after intravenous administration and high after nebulisation. Conversely, intravascular colistimethate sodium concentrations are low after nebulisation and high after intravenous administration; **f** and **g** total versus regional lung and plasma concentration–time profiles after the administration of 400 mg tobramycin by nebulisation or intravenously. The mean concentrations measured from four probes implemented in upper and lower lobes are represented in (**f**) and regional concentrations in (**g**). High lung and low plasma concentrations of nebulised tobramycin are evidenced by lung microdialysis; **h** distribution of tobramycin concentrations between proximal and distal airways immediately after the nebulisation of 600 mg in patients with cystic fibrosis. Aerosol concentrations in the central and more distal airways were computed using airway models reconstructed from computed tomography scans of patients with cystic fibrosis, in combination with computational fluid dynamic simulations. Proximal airways defined as bronchi with an internal diameter greater than 1 mm are represented as the tracheal bronchial tree, whereas distal airways are represented as lung parenchyma;** i** during the bronchoalveolar lavage performed to collect the epithelial lining fluid, the bronchoscope is heavily contaminated by the antibiotic deposited on bronchial walls during the nebulisation (red colour); **j** box plots showing higher epithelial lining fluid (ELF) than interstitial space fluid (ISF) tobramycin concentrations for nebulised tobramycin compared to intravenous (IV) tobramycin at a dose of 400 mg. The dots indicate the values that are outside the box plots. ELF concentrations are measured by bronchoalveolar lavage and ISF concentrations by lung microdialysis. **b**, **g** and **j** are reproduced from [13] and with permission of the publisher; **f** is reproduced from [12] with permission of the publisher; **h** and **i** are reproduced from reference [10] with permission of the publisher

Microdialysis is based on the principle of diffusion of molecules along their concentration gradient between two compartments. Lung microdialysis can measure antibiotic concentrations in the ISF. As lung microdialysis requires the insertion of probes within the lung parenchyma (Fig. 1b, c), it is an invasive technique essentially used in animals [12, 13]. It can be combined with intravascular microdialysis through the percutaneous insertion of an intravenous microdialysis catheter to assess systemic antibiotic concentrations. Principles, technique of implementation and method for lung microdialysis are described in Fig. 1a–g. Due to their respective dimensions (0.6 mm for the microdialysis catheter vs. 0.2 mm for an aerated alveolus), the microdialysis probe is in contact with several alveoli. In the aerated lung, membrane exchange will not take place with alveolar air. In the infected lung, alveoli are filled with fluid and cells allowing membrane exchanges and antibiotic concentrations in the dialysate reflect a mixture of interstitial, intracellular and alveolar concentrations. As VAP and inoculation experimental pneumonias are heterogeneous lung diseases, several probes are required to obtain an organ-wide overview unless the study is region-specific (Fig. 1c).

For lung pharmacokinetics studies, measurement of ISF concentrations is necessary as antibiotics exert their bactericidal effect at concentrations equal to five times the minimal inhibitory concentrations. Using lung microdialysis, ISF concentrations are higher than dialysate concentration owing to inability to achieve equilibrium between the ISF and the perfusion medium. The factor by which the concentrations are interrelated is termed “relative recovery”, which is dependent on the size and chemical properties of the antibiotic, the tissue coefficient and the perfusate flow rate. Hence, each microdialysis probe needs to be calibrated. The no net flux method is considered to be the gold standard for in vivo calibration. The principal disadvantage of this method is that it is time-consuming and requires steady-state conditions, which may be unattainable. However, the retrodialysis method may be used to calibrate the probe as it has been validated against the no net flux method [14].

Lung microdialysis to evaluate lung PK for nebulised antibiotics can have advantages over bronchoalveolar lavage and lung biopsies: measurement of unbound fraction, continuous sampling, regional assessment of antibiotic lung concentrations and no risk of bronchial contamination. In mechanically ventilated animals receiving nebulised antibiotics, it allows measuring the impact of changing ventilator modes and settings on lung concentrations.

Limitations of lung microdialysis include the need for its precise open thoracic surgical placement performed under direct vision to avoid bleeding, pneumothorax and parenchymal injury, and human application is limited to patients undergoing cardiac and thoracic surgery [15–23]. Lung microdialysis is challenging for sampling lipophilic antibiotics that bind to plastic surfaces (oxazolidinone, fluoroquinolones) [14]. Mobile lung may affect the probe stability and hence sample quality. The low dialysate volumes require optimised antibiotic assay techniques to measure analytes in such samples (reverse-phase high-performance liquid chromatography or liquid chromatography–tandem mass spectrometry, using either ultraviolet or fluorescence detectors). Large molecular size and highly protein-bound antibiotics like polymyxin B result in lower microdialysis recoveries.

### Lung microdialysis as a research tool to advance antimicrobial therapeutics for severe lung infections

#### Animal models and experimental design

Experimental studies using lung microdialysis are resource intensive and require specialist techniques handled by highly qualified researchers. Moreover, lung pharmacokinetic studies involving nebulised antibiotics for VAP require large animals undergoing prolonged mechanical ventilation in an experimental intensive care unit [7]. To provide optimal real-time data, the sampling frequency is high and costly. Therefore, the future design of multicentre experimental controlled trials is an attractive option for dealing with these constraints.

#### Pharmacokinetics of nebulized antibiotics

Various lung and nebulised antibiotic-specific factors may result in therapeutic failure and the emergence of multidrug-resistant organisms. Effective nebulised antibiotic therapy depends on the understanding and optimising of the antibiotic lung pharmacokinetics affected by various factors [24]. Lung microdialysis enables lung ISF sampling, providing an avenue to investigate the effect of different drugs, types of delivery devices, interfaces, and disease conditions. Combining in vivo pharmacokinetics with in vitro pharmacodynamic simulations could help optimise the nebulised antibiotic dosing regimen.

The technique of nebulisation is a key factor for optimising lung pharmacokinetics. In patients with VAP, it is recommended (1) to limit inspiratory flow turbulences by using specifically designed Y piece without sharp angles and changing ventilator settings during the nebulisation phase [6]: constant rather than decelerating inspiratory flow (volume controlled ventilation rather than pressure support); low rather than high respiratory frequency (12–15 breaths per minute); long rather than short inspiratory time (inspiratory time over total respiratory time = 50%); (2) to use dry inspiratory circuits; (3) to optimise the bolus effect by placing the nebuliser close to the ventilator; and (4) to avoid patient-ventilator asynchronies by administering a short-acting sedation (propofol) during the nebulisation phase are recommended [2, 25]. However, these recommendations are based on in vitro studies and require validation through lung microdialysis experiments to confirm their impact on nebulised antibiotic deposition in the lungs.

Furthermore, newer nebulisation therapies including repurposing of existing antibiotic formulations with systemic toxicity, novel antibiotics, bacteriophages and immunomodulators, can be thoroughly investigated using an incremental model of research prior to clinical applications. The use of lung microdialysis to understand how to optimise the nebulisation of polymyxins can inform dosing regimens. Nebulised polymyxin E and B are effective against extensive drug-resistant Gram-negative bacteria and are widely used worldwide for treating VAP caused by multidrug-resistant organisms [26]. Intravenous polymyxins have a limited penetration into the interstitial space fluid and a high systemic toxicity. Polymyxin E is a prodrug and requires an in vivo hydrolysis to release colistin, the active antibiotics. Polymyxin B has the potential for penetrating into the infected lung, but its high binding to proteins limits the effective penetration into the ISF [27]. Lung microdialysis can provide crucial data to inform optimised nebulised polymyxin dosing for effective therapy [27, 28].

### Lung microdialysis for developing theranostic tools

Pulmonary inflammation, aeration loss and regional blood flow can influence response to nebulised antibiotics. In vivo microdialysis has been used as a theranostic tool in the traumatic brain injury and brain tumours [29]. Lung microdialysis can potentially provide real-time insights into the dynamic biochemical changes in pneumonia by continuous monitoring of specific cytokines, metabolites and markers of inflammation/infection. Such experiments can inform timing and duration of nebulised antibiotic therapy, correlate antibiotic effects with changes in infection-specific proteomic biomarkers in lung ISF [28] and promote the development of theranostic tools to tailor treatment in the critically ill with VAP.

## Conclusion

In summary, lung microdialysis can emerge as a tool in advancing our understanding of nebulised antibiotics for VAP. It has the potential to enable dosing regimen optimisation based on precise target site pharmacokinetics instead of data from current sampling methods with their limitations (blood, epithelial lining fluid). Additionally, it has the potential to develop a theranostic tool for respiratory infections. With an increasing incidence of VAP caused by multidrug-resistant organisms in intensive care units, there is an urgent need to use lung microdialysis for investigating innovative treatment strategies; hence, lung microdialysis appears as a unique technique for investigating innovative treatment strategies, optimising nebulisation therapy and improving patient outcomes.

Addressing the high costs and complexity of the technique, the European Investigators Network for Nebulized Antibiotics in Ventilator-associated Pneumonia (ENAVAP) has taken a proactive step by forming the international research network for Lung Microdialysis applied to Nebulised Antibiotics (LUMINA). In a near future, the LUMINA network will promote multicentre experimental randomised controlled studies.

## Data Availability

Not applicable.

## Abbreviations

VAP: Ventilator-associated pneumonia
LUMINA: Lung microdialysis applied to nebulised antibiotics
ISF: Interstitial space fluid
ENAVAP: European Investigators Network for Nebulized Antibiotics in Ventilator-associated Pneumonia
